# Supplementary material for: Loss of TET2 and TET3 in regulatory T cells unleashes effector function
Source: Nat Commun. 2019 May 1;10:2011. doi: 10.1038/s41467-019-09541-y (PMC6494907; doi:10.1038/s41467-019-09541-y)
Supplement: Supplementary file 1 — Supplementary Information [file 41467_2019_9541_MOESM1_ESM.pdf]

Supplementary Information

Loss of TET2 and TET3 in regulatory T cells unleashes effector function

Yue et al.

This file includes:

Supplementary Figure1 to Figure13

Supplementary Table1 and Table2

Supplementary Figure 1

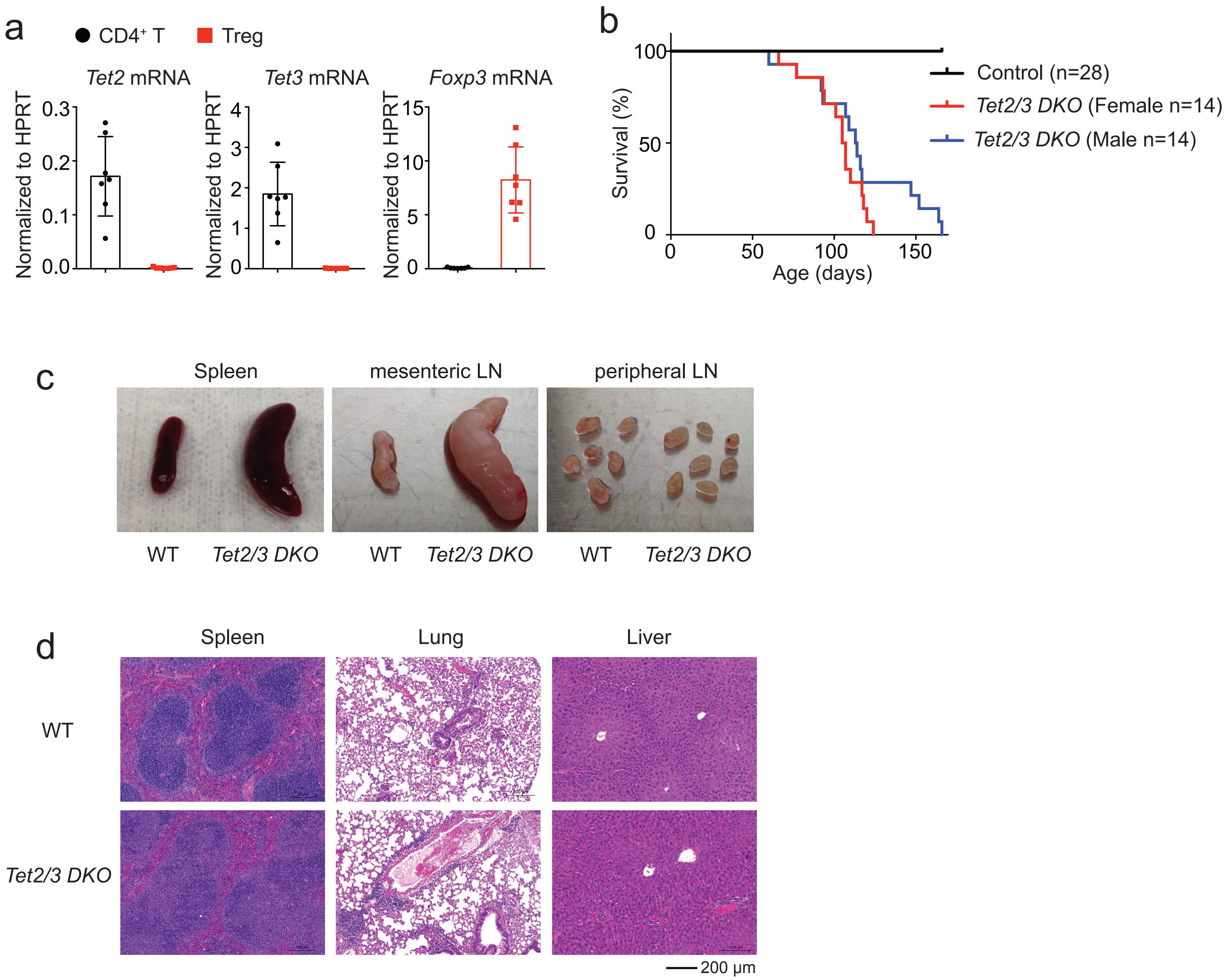

Supplementary Figure 1. Characterization of mice with Treg-specific deficiency of *Tet2* and *Tet3*.

a. Quantitative real-time PCR analysis of *Tet2*, *Tet3* and *Foxp3* expression level in CD4$^+$Foxp3$^-$ T cells (CD4$^+$ T) and CD4$^+$Foxp3$^+$ Treg cells (Treg) isolated from *Tet2/3$^{fl/fl}$Foxp3^{Cre}* DKO mice (13-16 weeks old). **b**. Survival curves for control WT (n=28) and *Tet2/3$^{fl/fl}$Foxp3^{Cre}* DKO mice separated into female and male groups (n=14 for each group). **c**. Representative pictures of spleen, mesenteric lymph nodes and peripheral lymph nodes (pLN) from WT and *Tet2/3$^{fl/fl}$Foxp3^{Cre}* DKO mice (14 weeks old). **d**. H&E staining of spleen, lung and liver of WT and *Tet2/3$^{fl/fl}$Foxp3^{Cre}* DKO mice (15-16 weeks old). Error bars show mean ± s.d. from at least three independent experiments.

Supplementary Figure 2

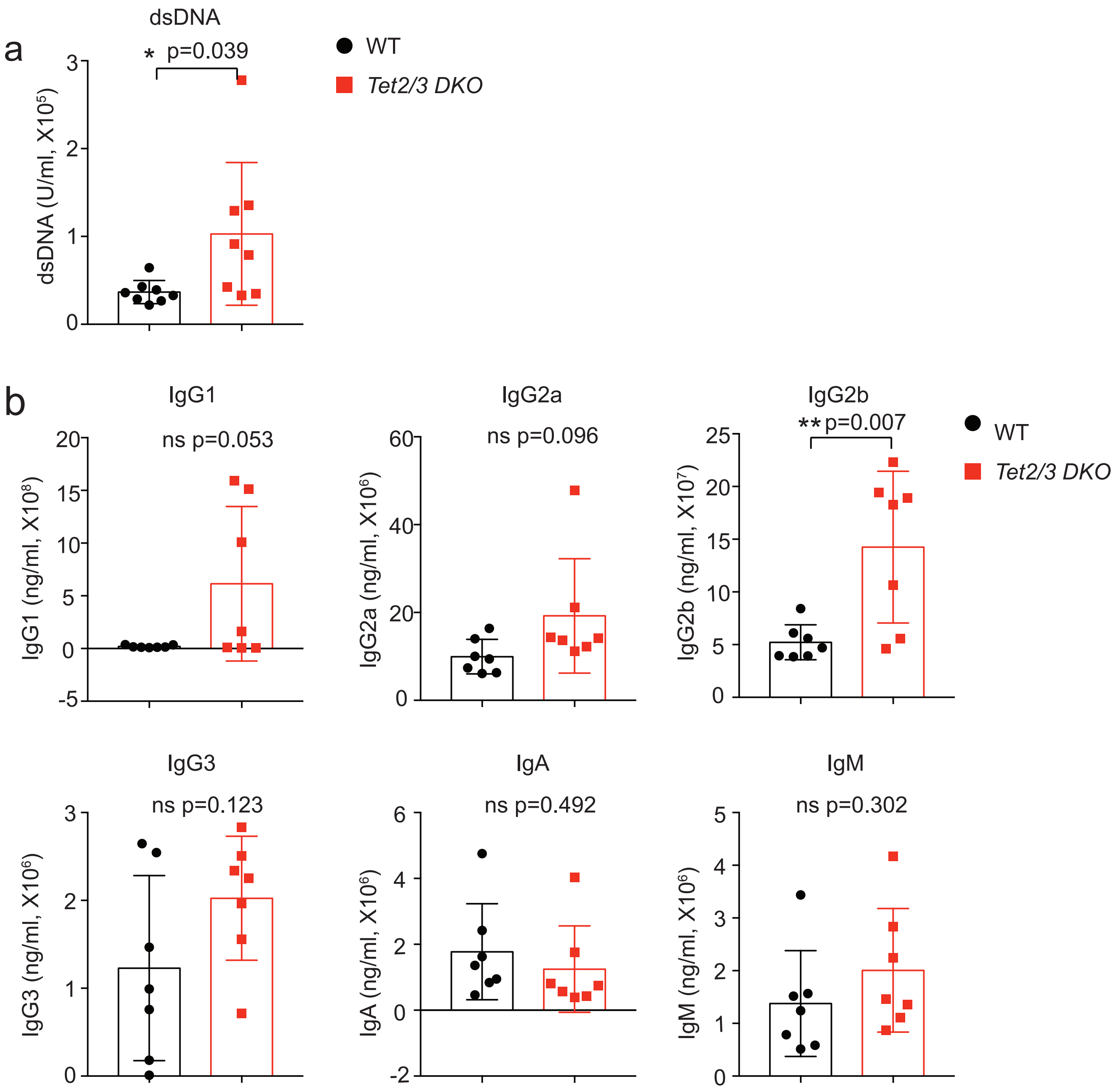

Supplementary Figure 2. Analysis of anti-dsDNA antibody and immunoglobulin levels in the serum isolated from *Tet2/3$^{fl/fl}$Foxp3^{Cre}* DKO mice.

a. Quantification of anti-dsDNA antibody in the serum of WT and *Tet2/3$^{fl/fl}$Foxp3^{Cre}* DKO mice (11-16 weeks old, WT n=8, *Tet2/3$^{fl/fl}$Foxp3^{Cre}* DKO mice n=7). **b**. Quantification of immunoglobulin IgG1, IgG2a, IgG2b, IgG3, IgA and IgM in the serum of WT and *Tet2/3$^{fl/fl}$Foxp3^{Cre}* DKO mice (11-16 weeks old, WT n=7, *Tet2/3$^{fl/fl}$Foxp3^{Cre}* DKO mice n=7). Error bars show mean ± s.d. from two independent experiments. Statistical analysis was performed using two-tailed unpaired student's t test (*P<0.05, **P<0.01).

Supplementary Figure 3

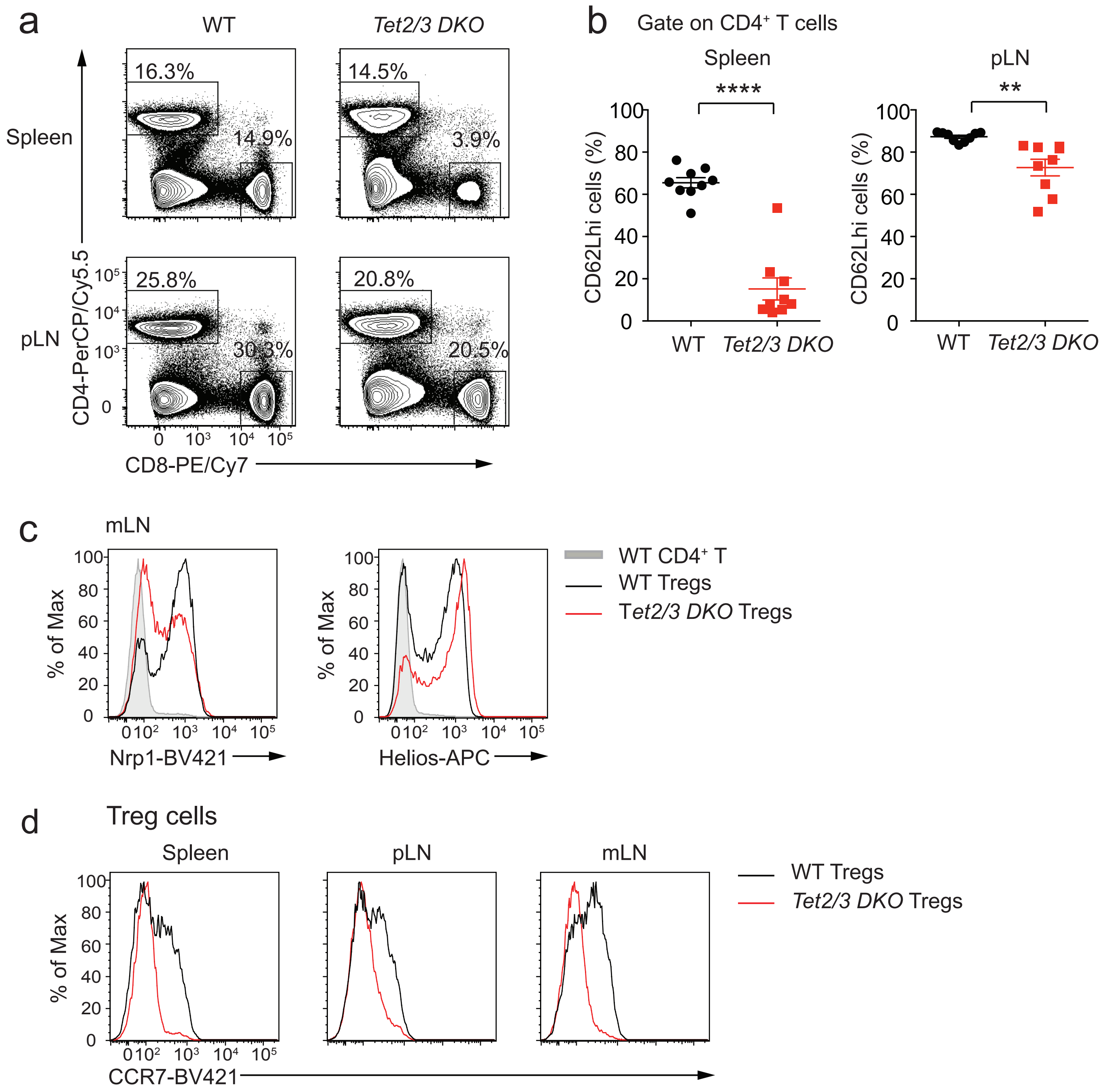

Supplementary Figure 3. Characterization of CD4$^+$ and CD8$^+$ T cell compartments and Treg cells in *Tet2/3*$^{fl/fl}$*Foxp3*^{Cre} DKO mice.

a. Representative flow cytometry analysis of CD4 and CD8 expression in spleen (*upper panels*) and pLN (*lower panels*) from WT and *Tet2/3*$^{fl/fl}$*Foxp3*^{Cre} DKO mice (13-16 weeks old). **b**. Quantification of the percentage of CD62L^{high} cells in CD4$^+$ T cells in spleen and pLNs from WT and *Tet2/3*$^{fl/fl}$*Foxp3*^{Cre} DKO mice (13-16 weeks old, see flow cytometry plots of Figure 1d). Statistical analysis was performed using two-tailed unpaired student's t test (**P<0.01, ***P<0.001). Error bars show mean ± s.d. from at least three independent experiments. **c**. Flow cytometry analysis of *Tet2/3*-deficient Treg cells (13-16 weeks old) from mLN for the expression of Nrp1 and Helios. Shown are WT CD4$^+$ T cells (shaded grey); WT Tregs (black line); DKO Tregs (red line). **d**. Flow cytometry analysis of *Tet2/3*-deficient Treg cells (13-16 weeks old) from spleen (*left*), pLN (*middle*) and mLN (*right*) for the expression of CCR7. Shown are WT Tregs (black line); DKO Tregs (red line).

Supplementary Figure 4

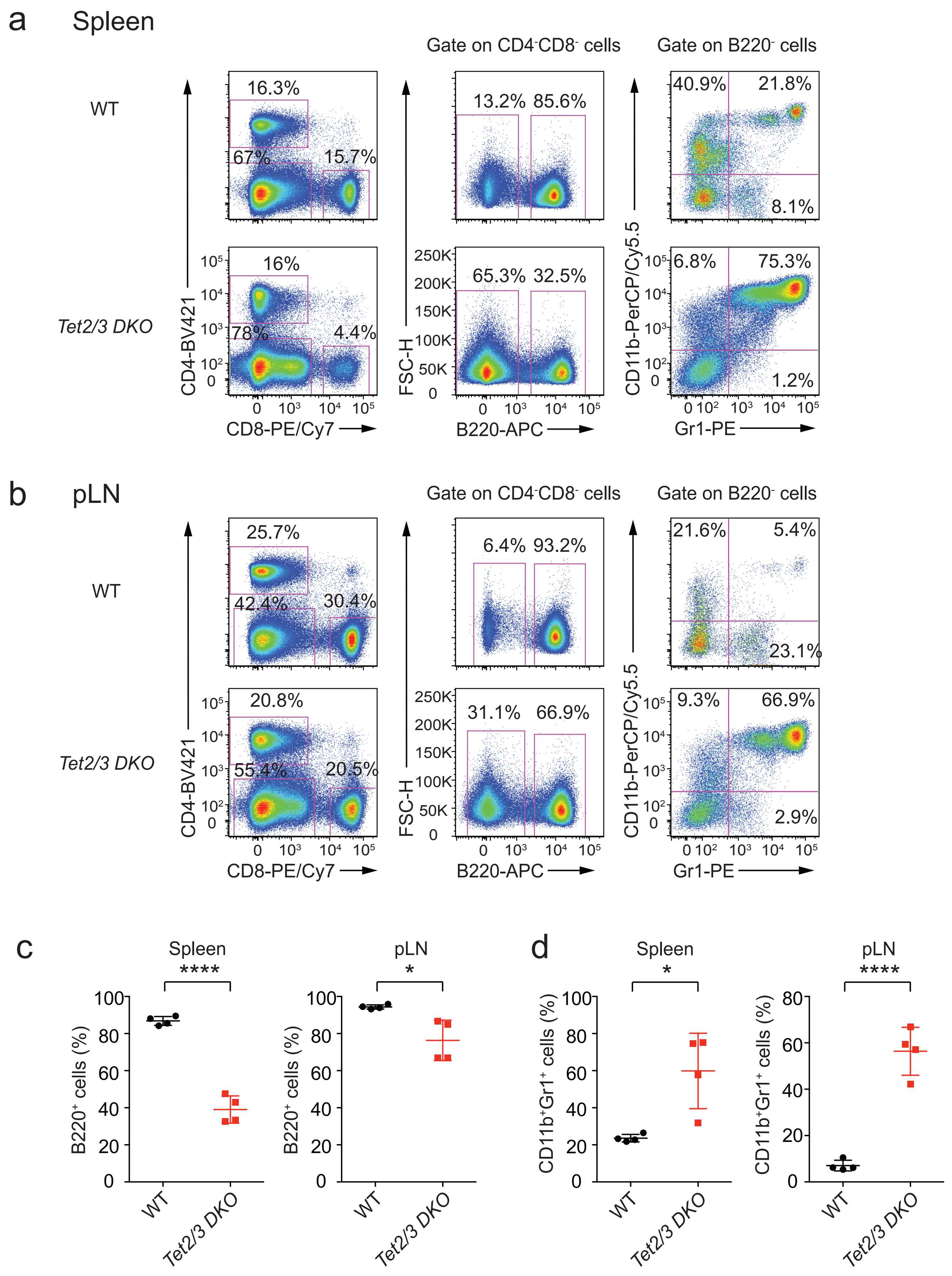

Supplementary Figure 4. Analysis of B cell and Myeloid cell lineages in *$Tet2/3^{fl/fl}Foxp3^{Cre}$ DKO* mice.

a-b. Flow cytometry staining of cells from spleen (**a**) and peripheral lymph nodes (pLN) (**b**) isolated from WT (*upper panels*) and *$Tet2/3^{fl/fl}Foxp3^{Cre}$ DKO* mice (*lower panels*) for the cell surface markers of CD4 and CD8 (*left panels*), B220 (*middle panels*, gated on $CD4^-CD8^-$ cells), CD11b and Gr1 (*right panels*, gated on $CD4^-CD8^-B220^-$ cells). **c-d**. Quantification of the percentage of $B220^+$ cells (**c**) and $CD11b^+Gr1^+$ cells (**d**) in spleen (*left panels*) and pLN (*right panels*) isolated from WT and *$Tet2/3^{fl/fl}Foxp3^{Cre}$ DKO* mice. Data are representative of four independent experiments. Statistical analysis was performed using two-tailed unpaired student's t test (*P<0.05, ****P<0.0001). Error bars show mean ± s.d. from three independent experiments.

Supplementary Figure 5

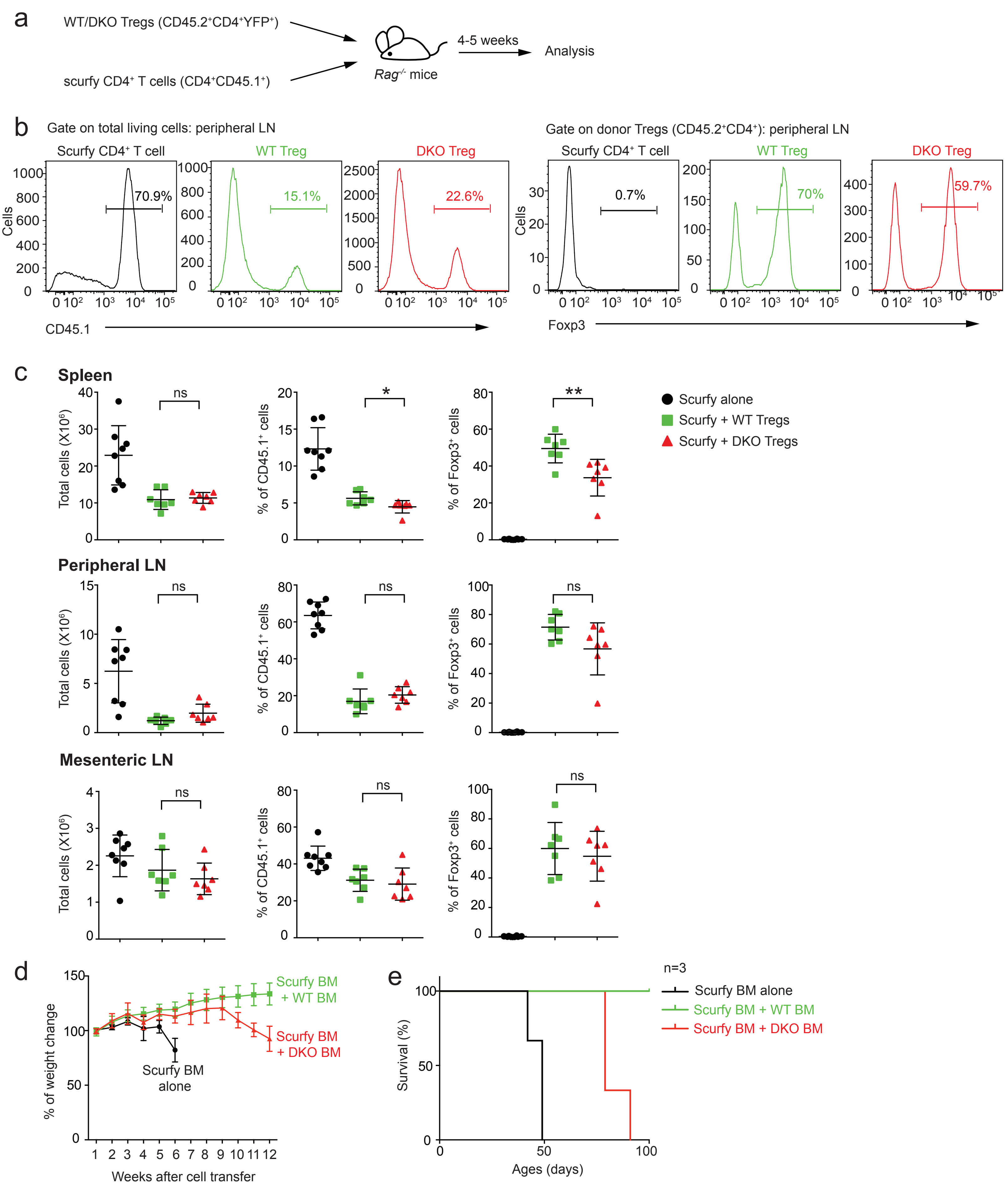

Supplementary Figure 5. ***Tet2/3 DKO*** **Treg cells can correct the** ***scurfy*** **phenotype in short term but not long-term assays.**

a. Schematic representation of the adoptive transfer experiment to assess the stability and suppressive function of Treg cells from 11-14 week-old *Tet2/3 DKO* mice *in vivo*. **b**. Representative histograms of $CD45.1^+$ cells (*left panel*) and $CD45.2^+CD4^+Foxp3^+$ cells (*right panel*) in peripheral lymph nodes from *Rag*-deficient mice 4-5 weeks after adoptive transfer of *scurfy* $CD4^+$ T cells alone or together with WT or DKO Treg cells. **c**. Graphs quantifying the total cellularity, percentage of $CD45.1^+$ cells and percentage of $Foxp3^+$ cells in transferred Treg cells from *Rag*-deficient mice, 4-5 weeks after adoptive transfer of scurfy $CD4^+$ T cells alone or together with WT or DKO Treg cells. WT and DKO Tregs show an equivalent ability to suppress the uncontrolled expansion of scurfy T cells. **d-e**. The percent weight change (**d**) and survival curves (**e**) after adoptive transfer of scurfy bone marrow cells, alone or together with WT or DKO bone marrow cells, into sub-lethally irradiated *Rag*-deficient mice (n=3). Statistical analysis was performed using two-tailed unpaired student's t test (*$P<0.05$, **$P<0.01$). Error bars show mean ± s.d.

Supplementary Figure 6

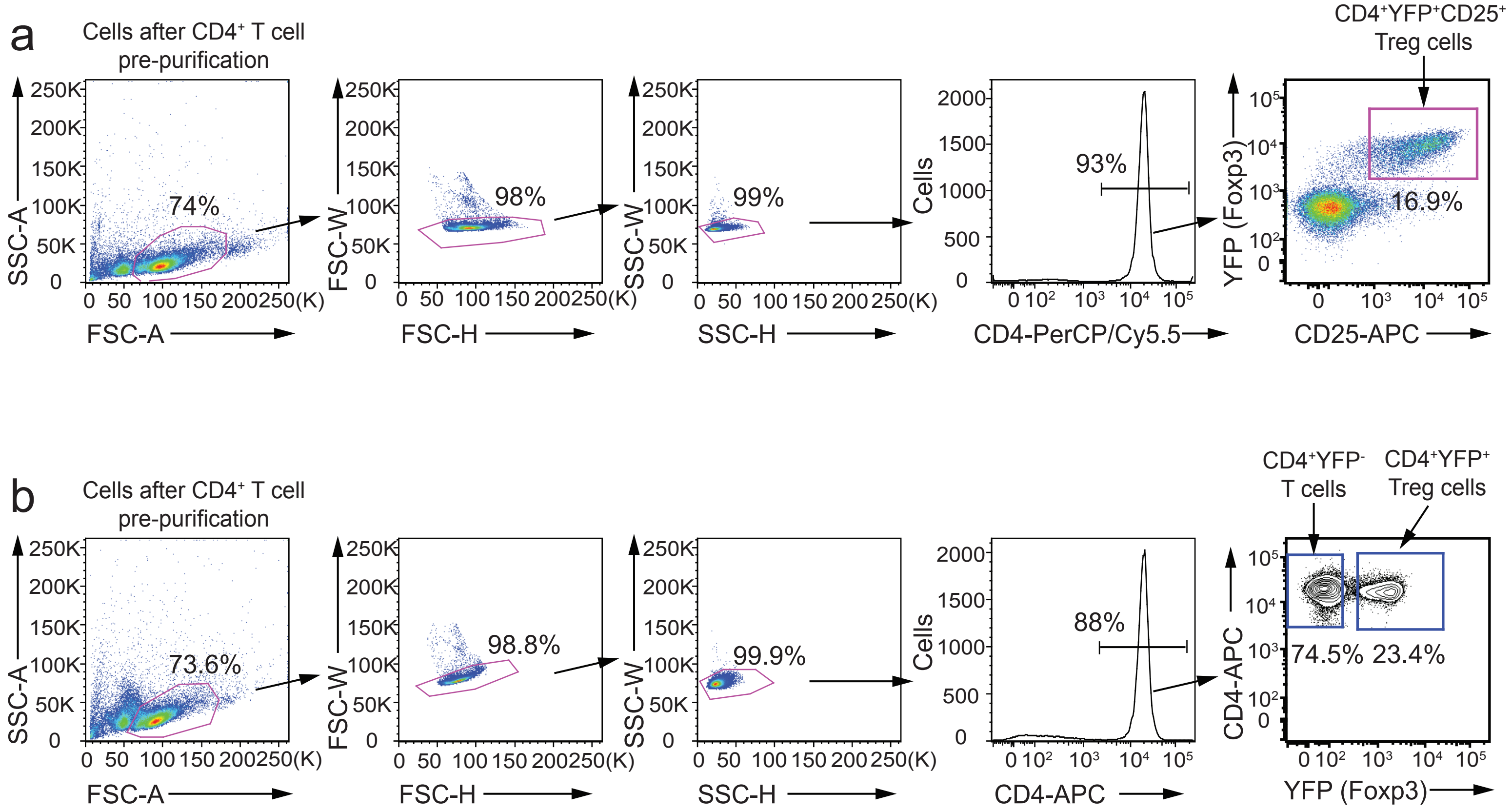

Supplementary Figure 6. Gating strategies used for cell sorting.

a. Gating strategy to sort CD4$^+$CD25$^+$YFP$^+$ Treg cells from WT and *Tet2/3*$^{fl/fl}$*Foxp3*^{Cre} DKO mice (8-10 weeks old) for DNA methylation analysis in Figure 3. **b**. Gating strategy to sort CD4$^+$YFP$^+$ Treg cells and CD4$^+$YFP$^-$ T cells from WT and *Tet2/3*$^{fl/fl}$*Foxp3*^{Cre} DKO mice (14 weeks old) for RNA-seq analysis in Figure 4 and in Supplementary Figure 8.

Supplementary Figure 7

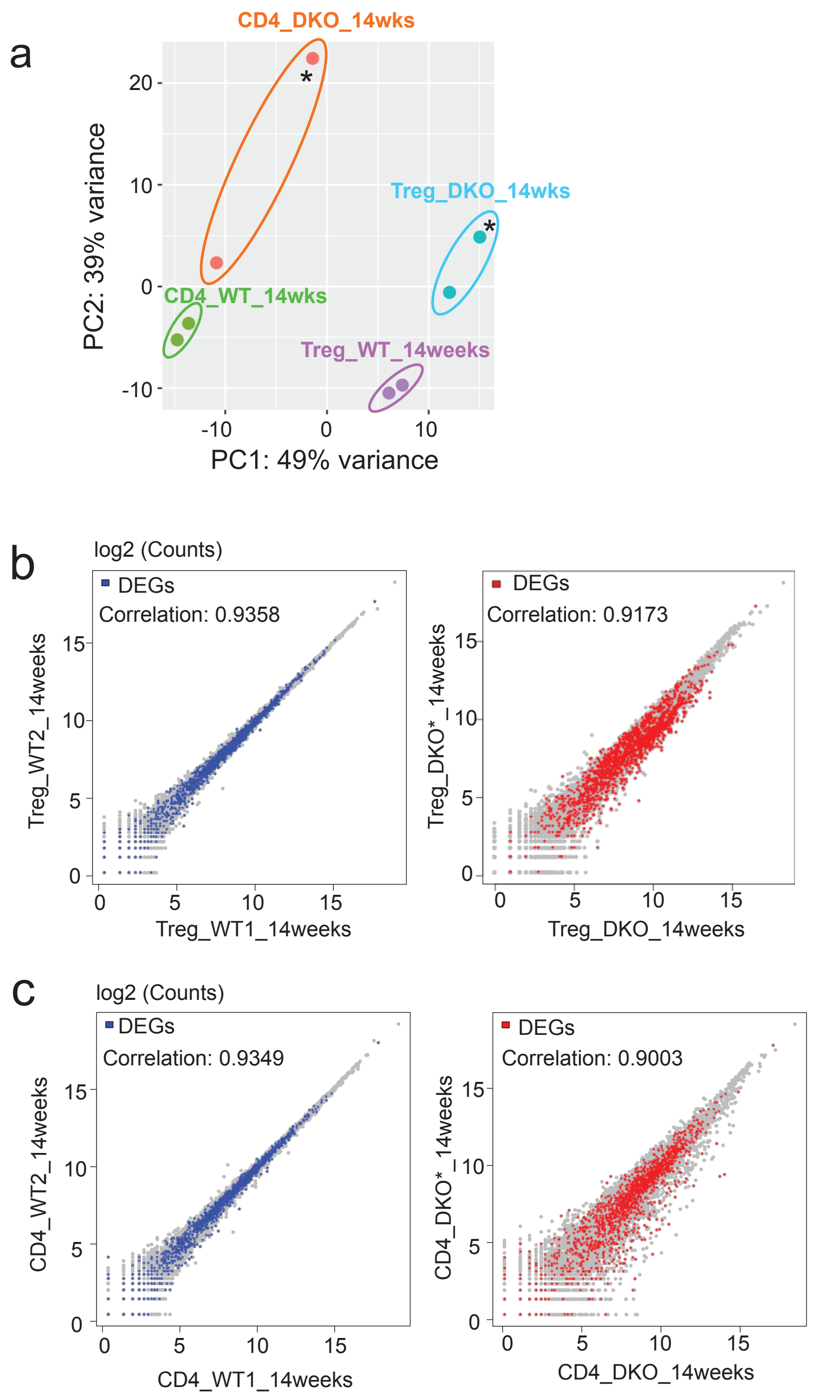

Supplementary Figure 7. PCA and correlation analysis for RNA-seq samples isoalted from pooled spleen and pLNs of WT or *Tet2/3$^{fl/fl}$Foxp3^{Cre} DKO* mice.

a. Principal component analysis (PCA) plot for replicate RNA-seq samples: comparison of WT and DKO Tregs and WT and DKO CD4^{+}Foxp3^{-} T cells from 14-weeks old mice. **b-c**. Scatter plots showing the correlation between replicates for Treg cells (**b**) and CD4^{+}Foxp3^{-} T cells (**c**) from 14-weeks old WT and *Tet2/3$^{fl/fl}$Foxp3^{Cre}* DKO mice.

Supplementary Figure 8

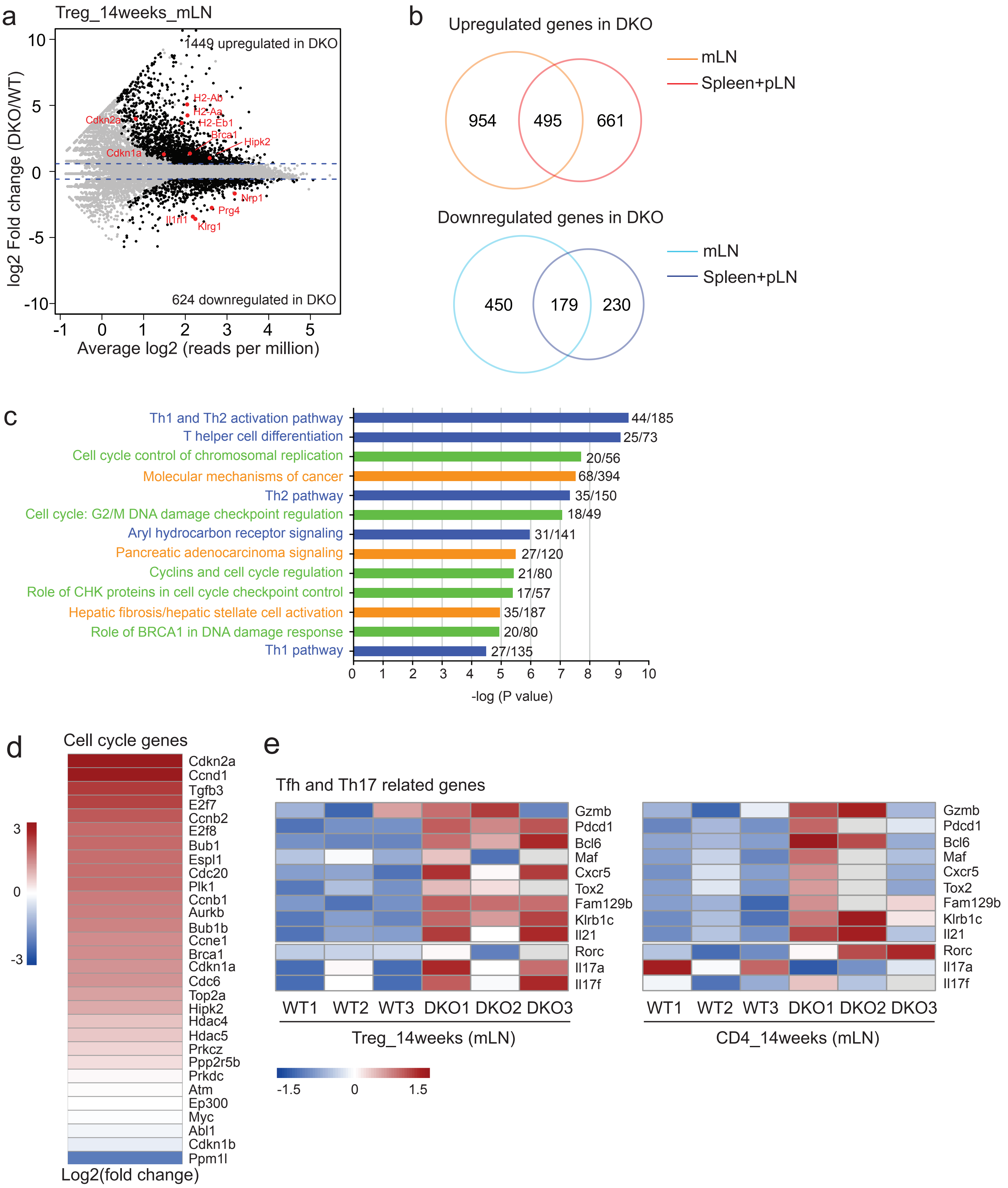

Supplementary Figure 8. RNA-seq analysis for Treg cells isolated from mLN of WT or *Tet2/3$^{fl/fl}$Foxp3^{Cre}* *DKO* mice.

a. Mean average (MA) plot of genes differentially expressed in *Tet2/3 DKO* Tregs (14-weeks old, isolated from mLN) relative to their expression in WT Treg cells. **b**. Overlap of differentially expressed genes that are upregulated (upper panel) or downregulated (lower panel) in *Tet2/3* DKO Treg cells isolated from either pooled spleen and pLNs or from mLN. **c**. IPA analysis of canonical pathways for differentially expressed genes in *Tet2/3 DKO* Treg cells isolated from mLN. Green, categories related to DNA repair, DNA damage and cell cycle; blue, categories related to immune cell function; orange, category related to cancer. **d**. Heatmap for the expression of selected genes encoding cell cycle regulators. The color gradient indicates Log2 Fold Change (DKO/WT). **e**. Heatmaps showing expression (row z score of log2 TPM values) of Tfh and Th17 related genes in WT and DKO Tregs (*left panel*) and WT and DKO CD4^{+}Foxp3^{-} cells (*right panel*).

Supplementary Figure 9

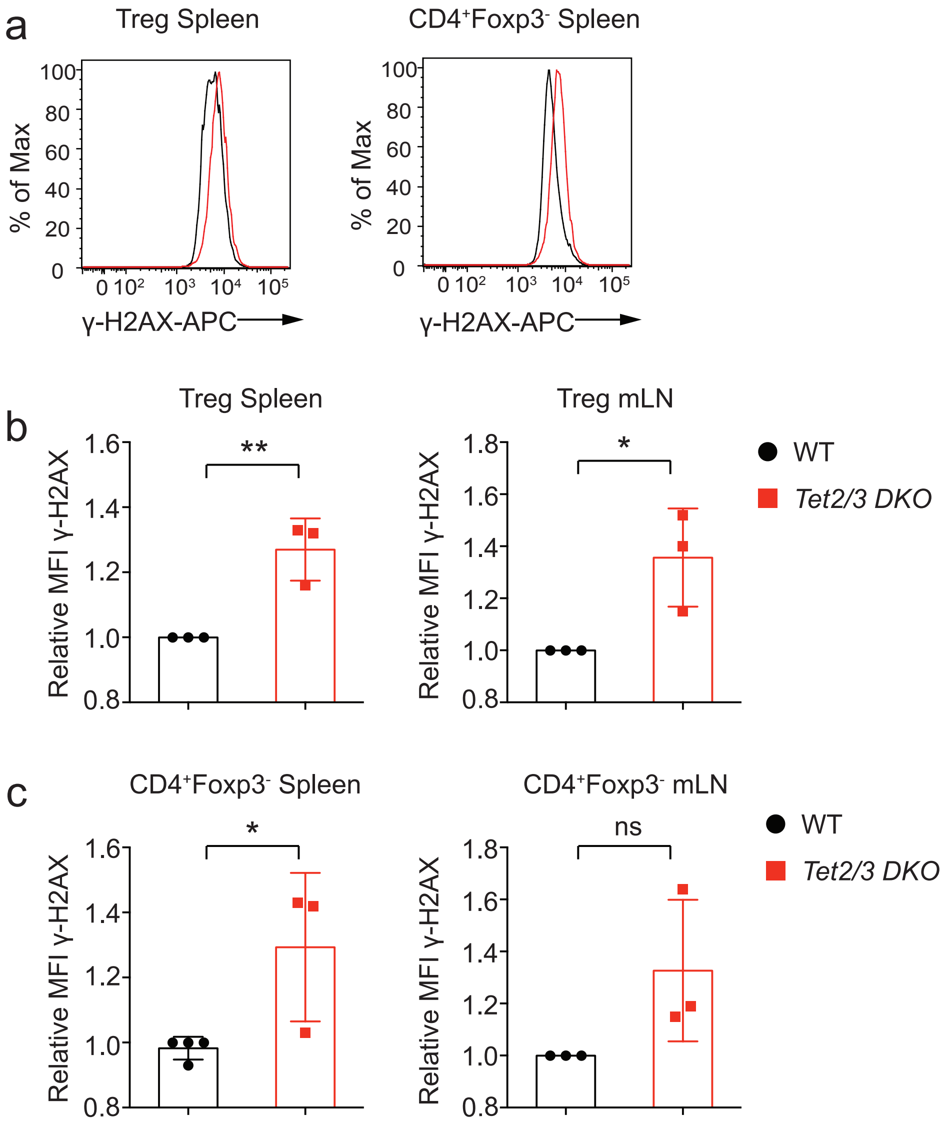

Supplementary Figure 9. *Tet2/3* DKO cells show increased level of DNA damage.

a. Representative flow cytometry analysis of γ-H2AX in Treg cells (*left panel*) and $CD4^+Foxp3^-$ T cells (*right panel*) in spleen isolated from WT and *Tet2/3*$^{fl/fl}$*Foxp3*^{Cre} DKO mice (13-16 weeks old) **b**. Relative MFI (mean fluorescent intensity) of γ-H2AX in Treg cells from WT and *Tet2/3*$^{fl/fl}$*Foxp3*^{Cre} DKO mice (13-16 weeks old) in Spleen (*left panel*) and mLN (*right panel*). **c**. Relative MFI of γ-H2AX in $CD4^+Foxp3^-$ cells from WT and *Tet2/3*$^{fl/fl}$*Foxp3*^{Cre} DKO mice (13-16 weeks old) in Spleen (*left panel*) and mLN (*right panel*). Error bars show mean ± s.d. from three independent experiments. Statistical analysis was performed using two-tailed unpaired student's t test (*P<0.05, **P<0.01, ns: not significant).

Supplementary Figure 10

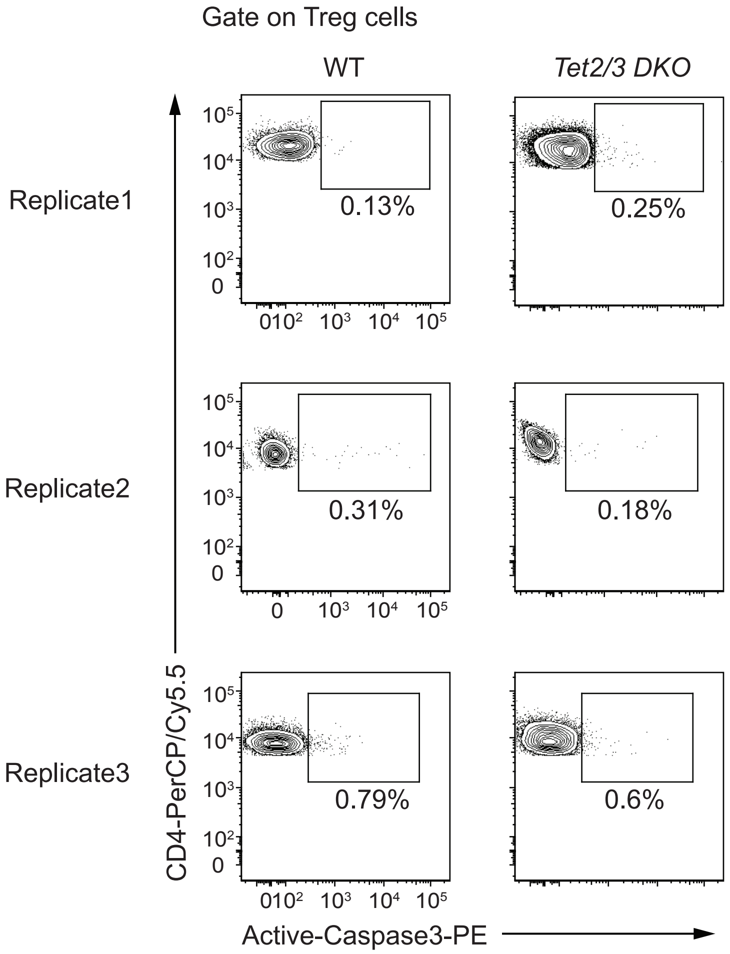

Supplementary Figure 10. Caspase 3 staining to evaluate the apoptosis in *Tet2/3* DKO Treg cells.

Flow cytometry staining of active caspase-3 in WT and *Tet2/3* DKO Treg cells using PE-conjugated anti-active Caspase-3 antibody. Data are from three independent experiments.

Supplementary Figure 11

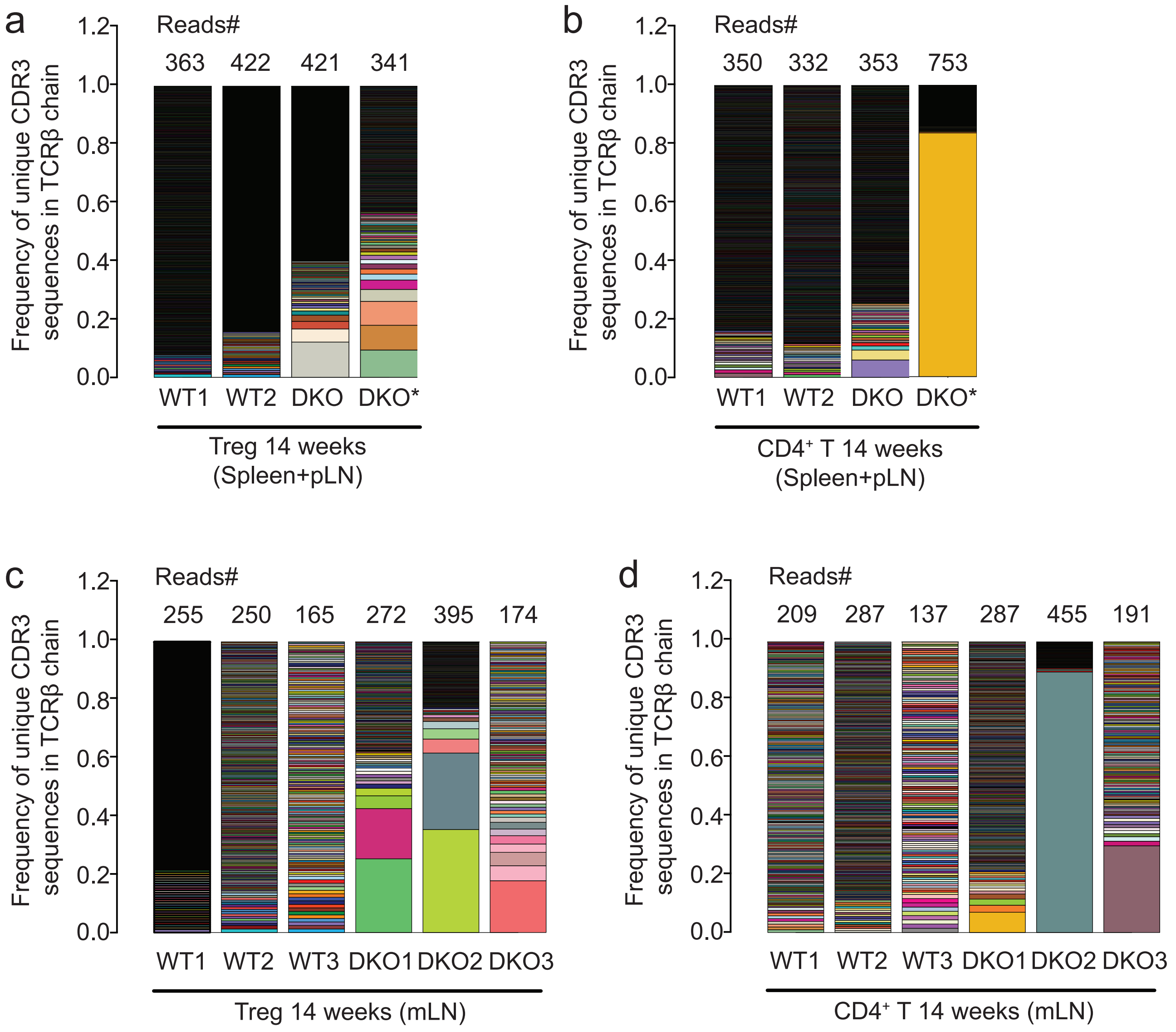

Supplementary Figure 11. *Tet2/3* DKO cells show increased T cell clonal expansion.

a-b. The frequency of reads (from the RNA-seq data of pooled spleen and pLNs) mapping to unique CDR3 sequences in TCRβ chain in WT and DKO Tregs from 14-week-old WT and *Tet2/3$^{fl/fl}$Foxp3^{Cre}* DKO mice (**a**), in WT and DKO CD4$^+$Foxp3$^-$ T cells from 14-week-old WT and *Tet2/3$^{fl/fl}$Foxp3^{Cre}* DKO mice (**b**). **c-d**. The frequency of reads (from the RNA-seq data of mLN) mapping to unique CDR3 sequences in TCRβ chain in WT and DKO Tregs from 14-week-old WT and *Tet2/3$^{fl/fl}$Foxp3^{Cre}* DKO mice (**c**), in WT and DKO CD4$^+$Foxp3$^-$ T cells from 14-week-old WT and *Tet2/3$^{fl/fl}$Foxp3^{Cre}* DKO mice (**d**). Each color represents a different TCRβ CDR3 sequence; the number of reads is shown on top of each graph.

Supplementary Figure 12

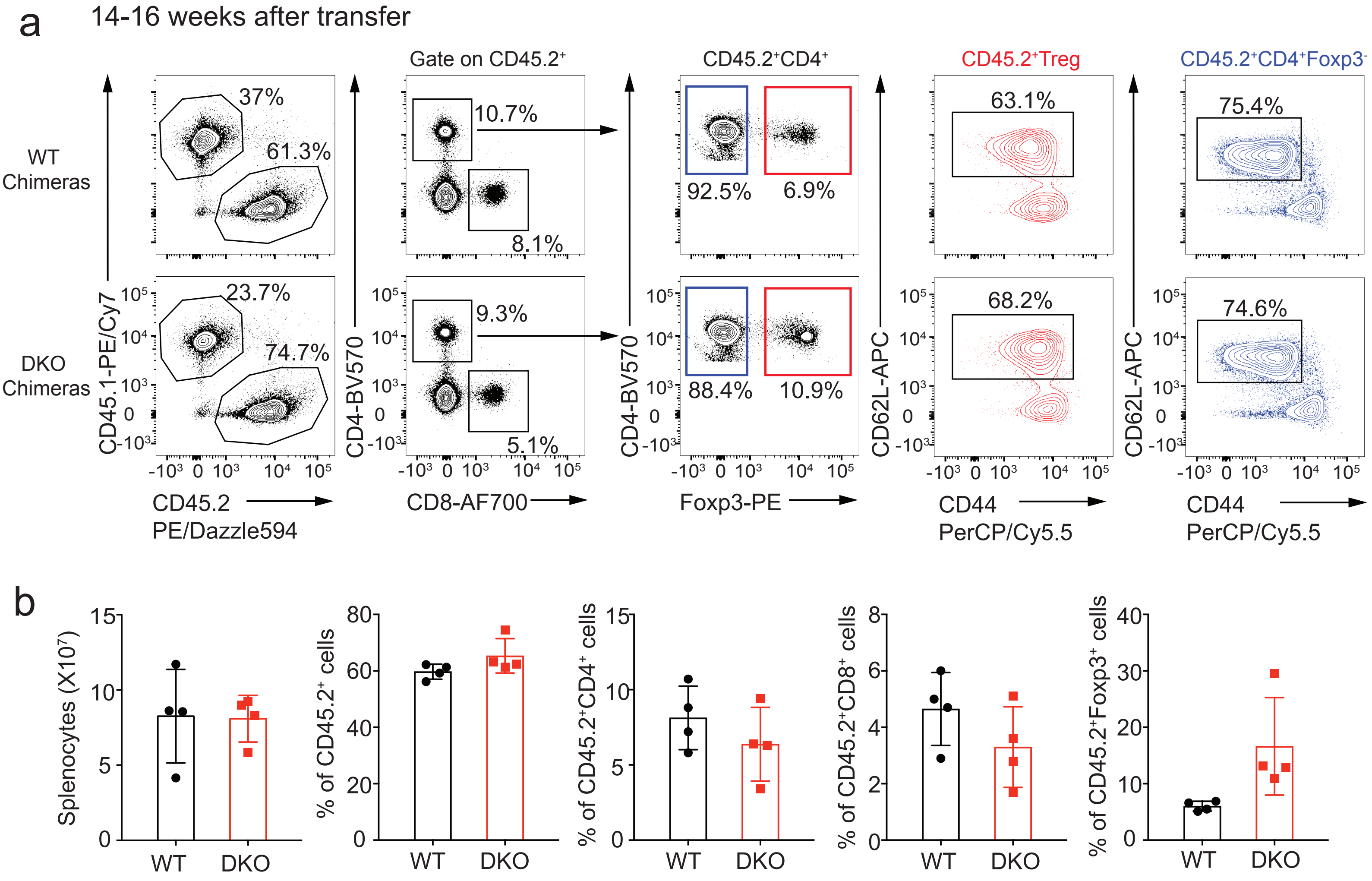

Supplementary Figure 12. Analyses of bone marrow chimeras at earlier time points, prior to splenomegaly.

a. Representative flow cytometry analysis for WT and DKO mixed bone marrow chimeras 14-16 weeks after transfer, before the development of splenomegaly. **b**. From left to right, the graphs show quantifications for the total number of splenocytes, the percentage of $CD45.2^+$ cells, the percentage of $CD4^+$ and $CD8^+$ cells within the $CD45.2^+$ cells and the percentage of $Foxp3^+$ cells within the $CD45.2^+CD4^+$ cells (n=4). Error bars show mean ± s.d. from three independent experiments.

Supplementary Figure 13

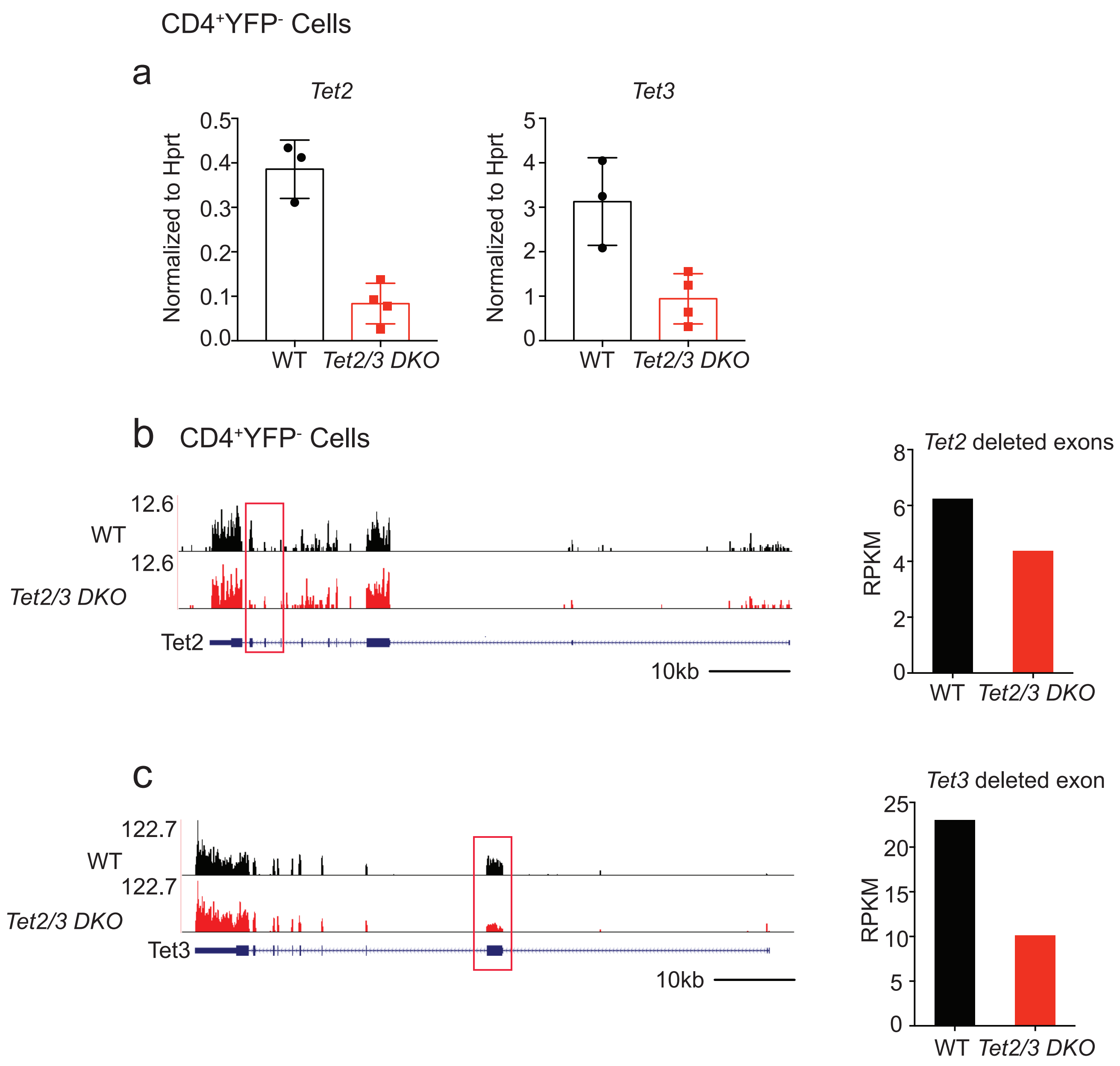

Supplementary Figure 13. Analyses of *Tet2* and *Tet3* expression at mRNA level in CD4^{+}YFP^{-} T cells from *Tet2/3*$^{fl/fl}$*Foxp3*^{Cre} DKO mice.

a. Quantitative real-time PCR analysis of *Tet2* and *Tet3* expression in CD4^{+}YFP^{-} cells from WT and *Tet2/3*$^{fl/fl}$*Foxp3*^{Cre} DKO mice (13-16 weeks old). Error bars show mean ± s.d. from three independent experiments. **b-c**. Genome browser tracks of *Tet2* and *Tet3* with the deleted exons highlighted in rectangles (*left panels*) and the number of normalized reads within the deleted exons (*right panels*).

Supplementary Table1. Hematological parameters in *Tet2/3* DKO mice

	WT	DKO	P value
WBC (10^3/µl)	9.66±1.62	8.66±0.95	0.3298
Neutrophil (103/ul)	1.91±0.30	2.98±0.84	0.0525
Lymphocyte (103/ul)	7.47±1.30	5.29±1.04	*, 0.0401
Monocyte (103/ul)	0.20±0.06	0.26±0.06	0.2248
Eosinophil (103/ul)	0.07±0.02	0.10±0.05	0.2508
Basophil (103/ul)	0.02±0.01	0.03±0.01	0.3202
RBC (106/ul)	9.75±0.25	9.48±0.33	0.2332
Hemoglobin (g/dL)	12.93±0.26	12.48±0.81	0.3291
Hematocrit (%)	46.05±0.91	44.50±1.82	0.1783
Platelet (103/ul)	1043.75±85.18	782±214.82	0.0641

Supplementary Table 2. Pearson Correlations between replicates for mLN RNA-seq

	Treg WT1	Treg WT2	Treg WT3
Treg WT1	1		
Treg WT2	0.942	1	
Treg WT3	0.942	0.943	1

	CD4 WT1	CD4 WT2	CD4 WT3
CD4 WT1	1		
CD4 WT2	0.943	1	
CD4 WT3	0.942	0.943	1

	Treg DKO1	Treg DKO2	Treg DKO3
Treg DKO1	1		
Treg DKO2	0.918	1	
Treg DKO3	0.922	0.917	1

	CD4 DKO1	CD4 DKO2	CD4 DKO3
CD4 DKO1	1		
CD4 DKO2	0.913	1	
CD4 DKO3	0.926	0.918	1
