# Supplementary material for: Loss of TET2 and TET3 in regulatory T cells unleashes effector function
Source: Nat Commun. 2019 May 1;10:2011. doi: 10.1038/s41467-019-09541-y (PMC6494907; doi:10.1038/s41467-019-09541-y)
Supplement: Supplementary file 3 — Description of Additional Supplementary Files [file 41467_2019_9541_MOESM3_ESM.pdf]

Description of Additional Supplementary Files

File Name: Supplementary Data 1
Description: Altered Treg signature genes in 14 week-old Tet2/3 DKO Tregs

File Name: Supplementary Data 2
Description: Differentially expressed genes involved in different pathways (pooled spleen and pLNs)

File Name: Supplementary Data 3
Description: Differentially expressed genes involved in different pathways (mLNs)

File Name: Supplementary Data 4
Description: CDR3 sequences for Treg cells and CD4+ T cells from periphery

File Name: Supplementary Data 5
Description: CDR3 sequences for Treg cells and CD4+ T cells from mLNs
