# Supplementary material for: Loss of TET2 and TET3 in regulatory T cells unleashes effector function
Source: Nat Commun. 2019 May 1;10:2011. doi: 10.1038/s41467-019-09541-y (PMC6494907; doi:10.1038/s41467-019-09541-y)
Supplement: Supplementary file 9 — Reporting Summary [file 41467_2019_9541_MOESM9_ESM.pdf]

nature research

Corresponding author(s): Anjana Rao

Reporting Summary

Nature Research wishes to improve the reproducibility of the work that we publish. This form provides structure for consistency and transparency in reporting. For further information on Nature Research policies, see Authors & Referees and the Editorial Policy Checklist.

Statistical parameters

When statistical analyses are reported, confirm that the following items are present in the relevant location (e.g. figure legend, table legend, main text, or Methods section).

n/a	Confirmed	
☐	☒	The exact sample size (*n*) for each experimental group/condition, given as a discrete number and unit of measurement
☐	☒	An indication of whether measurements were taken from distinct samples or whether the same sample was measured repeatedly
☐	☒	The statistical test(s) used AND whether they are one- or two-sided<br>*Only common tests should be described solely by name; describe more complex techniques in the Methods section.*
☐	☒	A description of all covariates tested
☒	☐	A description of any assumptions or corrections, such as tests of normality and adjustment for multiple comparisons
☐	☒	A full description of the statistics including central tendency (e.g. means) or other basic estimates (e.g. regression coefficient) AND variation (e.g. standard deviation) or associated estimates of uncertainty (e.g. confidence intervals)
☒	☐	For null hypothesis testing, the test statistic (e.g. *F*, *t*, *r*) with confidence intervals, effect sizes, degrees of freedom and *P* value noted<br>*Give P values as exact values whenever suitable.*
☒	☐	For Bayesian analysis, information on the choice of priors and Markov chain Monte Carlo settings
☒	☐	For hierarchical and complex designs, identification of the appropriate level for tests and full reporting of outcomes
☒	☐	Estimates of effect sizes (e.g. Cohen's *d*, Pearson's *r*), indicating how they were calculated
☐	☒	Clearly defined error bars<br>*State explicitly what error bars represent (e.g. SD, SE, CI)*

Our web collection on statistics for biologists may be useful.

Software and code

Policy information about availability of computer code

Data collection

Provide a description of all commercial, open source and custom code used to collect the data in this study, specifying the version used OR state that no software was used.

Data analysis

Flow Cytometry: Data were collected using Diva software on BD LSR II, LSR Fortessa. All data were analyzed using Flow Jo software (Tree Star, version 9.7.6 and 10.3.0)

Graphs and statistics: GraphPad Prism Version 7 was used to plot graphs, mean with standard deviation was shown on each graph. The statistical significance was performed using two-tailed unpaired student's t test.

RNA-seq analysis: RNA-seq data were mapped against the UCSC mouse genome mm9 using TopHat2 (v2.1.1) with the following parameters "-p 16 -N 2 --max-multihits 1 --read-gap-length 1 --transcriptome-index " and the RefSeq gene annotation was obtained from the UCSC genome Bioinformatics database. The number of reads mapping to each gene was counted using featureCounts (subread-1.4.3-p1) with the following parameters "-g gene_name –s 2". Differentially expressed genes (DEGs) between WT and Tet2/3 DKO cell types were determined using the Bioconductor package DESeq2 with adjusted P value < 0.05 and a fold change threshold of > 1.5 or <0.67. And genes with total counts < 1 in the sum of all conditions were removed from the analysis. Canonical pathway analysis was performed using Ingenuity Pathway Analysis software (Qiagen, license for La Jolla Institute).

TCR-seq analysis: TCR sequences were retrieved from RNA-Seq data sets, and the frequency of TCRβ chain clonotypes (CDR3 regions) was determined using MiXCR (mixcr-1.7-2.1) package for RNA-seq analysis with the default parameters "align –l TCR –s mmu –p rnaseq -

OallowPartialAlignments=true". Two rounds of contig assembly were performed by employing the "assemblePartial" function; extension of incomplete TCR CDR3s with uniquely determined V and J genes using germline sequences was done using the "extendAlignments" function; assembly and export of the clonotypes was performed using the "assemble" and the "exportClones" (--preset min -fraction -targets -vHits -dHits -jHits -vAlignments -dAlignments -jAlignments) functions, respectively.

For manuscripts utilizing custom algorithms or software that are central to the research but not yet described in published literature, software must be made available to editors/reviewers upon request. We strongly encourage code deposition in a community repository (e.g. GitHub). See the Nature Research guidelines for submitting code & software for further information.

Data

Policy information about availability of data

All manuscripts must include a data availability statement. This statement should provide the following information, where applicable:

- Accession codes, unique identifiers, or web links for publicly available datasets
- A list of figures that have associated raw data
- A description of any restrictions on data availability

Our RNA-seq data are available with the following accession number: GSE113694

Field-specific reporting

Please select the best fit for your research. If you are not sure, read the appropriate sections before making your selection.

☒ Life sciences ☐ Behavioural & social sciences ☐ Ecological, evolutionary & environmental sciences

For a reference copy of the document with all sections, see nature.com/authors/policies/ReportingSummary-flat.pdf

Life sciences study design

All studies must disclose on these points even when the disclosure is negative.

Sample size	Sample size was determined by previous studies or pilot studies
Data exclusions	No data was excluded from the analysis
Replication	All attempts at replication were successful
Randomization	Randomization was not applicable in this study. Sex and age matched animals were used for experimental and control groups
Blinding	Blinding is not relevant to the study since randomization was not applicable

Reporting for specific materials, systems and methods

Materials & experimental systems

n/a	Involved in the study	
☒	☐	Unique biological materials
☐	☒	Antibodies
☒	☐	Eukaryotic cell lines
☒	☐	Palaeontology
☐	☒	Animals and other organisms
☒	☐	Human research participants

Methods

n/a	Involved in the study	
☒	☐	ChIP-seq
☐	☒	Flow cytometry
☒	☐	MRI-based neuroimaging

Antibodies

Antibodies used	For analysis of T cell compartments and Treg cell features, single-cell suspensions were stained with anti-mouse antibodies against the following (Clone name, conjugated fluorescence, dilution, manufacturer and catalog number shown in brackets): CD4 (RM4-5, PerCP-Cy5.5, 1:200, Biolegend, #100540; GK1.5, APC, 1:200, Biolegend, #100412; RM4-5, BV570, 1:200, Biolegend, #100541; GK1.5, BV421, 1:200, Biolegend, #100437), CD8 (53-6.7, PE-Cy7, 1:200, Biolegend, #100722; 53-6.7, AF700, 1:200, Biolegend, #100730), CD62L (MEL-14, BV421, 1:400, Biolegend, #104436; MEL-14, APC, 1:200, Biolegend, #104412), CD25

(PC61, APC, 1:200, Biolegend, #102012), CD44 (IM7, PE, 1:200, Biolegend, #103008; IM7, PerCP-Cy5.5, 1:200, Biolegend, #103032), CD45.1 (A20, PE-Cy7, 1:200, Biolegend, #110730), CD45.2 (104, PE-Dazzle594, 1:200, Biolegend, #109846; 104, BV421, 1:200, Biolegend, #109831), Nrp1 (3E12, BV421, 1:200, Biolegend, #145209), ICOS (C398.4A, AF488, 1:200, Biolegend, #313514), CD103 (2E7, FITC, 1:200, Biolegend, #121419), GITR (YGITR765, PE-Cy7, 1:200, Biolegend, #120222), PD1 (29F.1A12, PE-Cy7, 1:200, Biolegend, #135216), CD127 (A7R34, BV421, 1:200, Biolegend, #135027), CD69 (H1.2F3, APC, 1:200, Biolegend, #104514), B220 (RA3-6B2, APC, 1:200, Biolegend, #103212), Gr1 (RB6-8C5, PE, 1:400, Biolegend, #108408), CD11b (M1/70, PerCP-Cy5.5, 1:200, Biolegend, #101228), CCR7 (4B12, BV421, 1:100, Biolegend, #120119). Anti-mouse CCR7 staining was performed at 37°C for 30min. For analysis of Tfh and germinal center B cells, single-cell suspensions were stained with anti-mouse antibodies TCRβ (H57-597, APC, 1:200, Biolegend, #109212), CD19 (6D5, APC, 1:200, Biolegend, #115512), CXCR5 (L138D7, BV421, 1:100, Biolegend, #145511), PD1 (29F.1A12, PE-Cy7, 1:200, Biolegend, #135216), GL7 (GL7, Pacific Blue, 1:200, Biolegend, #144613) and CD95 (Fas, Jo2, PE-Cy7, 1:200, BD Biosciences, #557653). For intracellular staining, cells were surface-stained and then stained with anti-Foxp3 (FJK-16S, PE, 1:100, eBioscience, #12-5773-82), anti-active caspase-3 (C92-605, PE, 20µl per assay, BD Biosciences, #550821), anti-Helios (22F6, APC, 1:100, Biolegend, #137221), anti-CTLA4 (UC104.89, APC, 1:100, Biolegend, #106309) and anti-H2A.X phosphorylated (Ser139, 2F3, APC, 1:100, Biolegend, #613415) antibodies using the Foxp3 Fixation/ Permeabilization kit (eBioscience, #00-5523-00) and analysed by flow cytometry on LSR-II and LSR Fortessa.

Validation

Describe the validation of each primary antibody for the species and application, noting any validation statements on the manufacturer's website, relevant citations, antibody profiles in online databases, or data provided in the manuscript.

Animals and other organisms

Policy information about studies involving animals; ARRIVE guidelines recommended for reporting animal research

Laboratory animals

B6.129(Cg)-Foxp3tm4(YFP/icre)Ayr/J (Foxp3Cre, strain 016959), B6.SJL-PtprcaPepcb/BoyJ (CD45.1 congenic mice, strain 002014), B6.129S7-Rag1tm1Mom/J (Rag1-/- mice, strain 002216) and B6.Cg-Foxp3sf/J (B6-scurfy mice, strain 004088) were obtained from Jackson Laboratory. Tet2fl/flTet3fl/flFoxp3Cre mice were generated in our laboratory by crossing Tet2fl/flTet3fl/f with Foxp3Cre mice. For the fate-mapping experiments, Tet2+/flTet3fl/flFoxp3Cre or Tet2fl/flTet3+/flFoxp3Cre female mice were crossed with Tet2fl/flTet3fl/flRosa26-YFP+ male mice to generate Tet2fl/flTet3fl/flFoxp3CreRosa26-YFP+ male mice for further analysis. All mice were on the B6 background and maintained in a specific pathogen-free animal facility in the La Jolla Institute for Immunology. Age of the mice used for each experiment was stated in the figure legends. All cell or mouse irradiation procedures were performed using RS2000 Biological Irradiator (Rad Source Technologies, Inc.) All animal procedures were reviewed and approved by the Institutional Animal Care and Use Committee of the La Jolla Institute for Immunology and were conducted in accordance with institutional guidelines

Wild animals

Provide details on animals observed in or captured in the field; report species, sex and age where possible. Describe how animals were caught and transported and what happened to captive animals after the study (if killed, explain why and describe method; if released, say where and when) OR state that the study did not involve wild animals.

Field-collected samples

For laboratory work with field-collected samples, describe all relevant parameters such as housing, maintenance, temperature, photoperiod and end-of-experiment protocol OR state that the study did not involve samples collected from the field.

Flow Cytometry

Plots

Confirm that:

☒ The axis labels state the marker and fluorochrome used (e.g. CD4-FITC).

☒ The axis scales are clearly visible. Include numbers along axes only for bottom left plot of group (a 'group' is an analysis of identical markers).

☒ All plots are contour plots with outliers or pseudocolor plots.

☒ A numerical value for number of cells or percentage (with statistics) is provided.

Methodology

Sample preparation

Single-cell suspensions were prepared from spleen, peripheral lymph nodes and mesenteric lymph nodes by mashing the organs through a 70µm cell strainer for staining or cell sorting.

Instrument

The cell sorting was done using BD FACS Aria; flow cytometry analysis was done using BD LSR II and Fortessa.

Software

The data were collected using FACS Diva software and analyzed using FlowJo Software (Version 9.7.6 and version 10.3.0)

Cell population abundance

The samples used for RNA-seq library construction were sorted and the purity is >99% determined by post-sort flow cytometry analysis; the purity of samples purified using dynabeads is >98-98.5% determined by flow cytometry analysis.

Gating strategy

Sorting for samples used for RNA-seq: CD4+ T cells were first enriched using untouched dynabeads mouse CD4 T cell kit, then sorted by gating on lymphocytes in FSC/SSC gate followed by exclusion of doublet, then gate on CD4+ T cells, and sort for CD4 +YFP(Foxp3)+ Treg cells and CD4+YFP(Foxp3)- T cells.

Sorting for samples used for amplicon-based bisulfite sequencing: CD4+ T cells were first enriched using untouched dynabeads mouse CD4 T cell kit, then sorted by gating on lymphocytes in FSC/SSC gate followed by exclusion of doublet, then gate on CD4+ T cells, followed by YFP (Foxp3)+ CD25+ cells;

Treg cells: lymphocytes in FSC/SSC gate followed by exclusion of doublet, then gate on CD4+YFP(Foxp3)+ cells

Tfh cells: lymphocytes in FSC/SSC gate followed by exclusion of doublet, then gate on CD4+TCRbeta+ T cells, Tfh cells were defined as CXCR5high PD-1high population;

Germinal Center B cells: lymphocytes in FSC/SSC gate followed by exclusion of doublet, then gate on CD19+ B cells, germinal center B cells were defined as GL7+CD95+ population;

CD45.1 and CD45.2 congenic markers were used to distinguish WT and DKO cells in mixed bone marrow chimeras; or distinguish transferred from host cells in CD4+ T cell transfer experiments.

☒ Tick this box to confirm that a figure exemplifying the gating strategy is provided in the Supplementary Information.
